# Digital Entry-Level Education in Physiotherapy: a Commentary to Inform Post-COVID-19 Future Directions

**DOI:** 10.1007/s40670-021-01439-z

**Published:** 2021-11-04

**Authors:** Giacomo Rossettini, Andrea Turolla, Bjorg Gudjonsdottir, Eleni Kapreli, Beate Salchinger, Geert Verheyden, Alvisa Palese, Andrea Dell’Isola, John Xerri de Caro

**Affiliations:** 1 School of Physiotherapy, University of Verona, Via Bengasi 4, 37134 Verona, Italy; 2 Laboratory of Rehabilitation Technologies, San Camillo IRCCS Srl, Via Alberoni 70, 30126 Venice, Italy; 3 Department of Physical Therapy, School of Health Sciences, University of Iceland, Stapi At Hringbraut, 101 Reykjavík, Iceland; 4 Department of Physiotherapy, School of Health Sciences, University of Thessaly, 3rd km Old National Road Lamia-Athen, 35100 Lamia, Greece; 5 Institute of Physiotherapy, FH JOANNEUM, Eggenberger Allee 13, 8020 Graz, Austria; 6 Department of Rehabilitation Sciences, KU Leuven, University of Leuven, Tervuursevest 101, 3001 Leuven, Belgium; 7 Department of Medical Sciences, School of Nursing, University of Udine, Viale Ungheria 20, 33100 Udine, Italy; 8 Department of Clinical Sciences Orthopaedic, Faculty of Medicine, Lund University, Entrégatan 8, 22100 Lund, Sweden; 9 Department of Clinical Sciences Orthopaedics, Clinical Epidemiology Unit, Lund University, Lund, Sweden; 10 Department of Physiotherapy, Faculty of Health Sciences, University of Malta, Triq Dun Karm, L-Imsida, Msida, 2090 MSD Malta

**Keywords:** Coronavirus disease 2019, COVID-19, Digital education, Student, Physiotherapy, Entry-level

## Abstract

Currently, the coronavirus disease 2019 (COVID-19) severely influences physiotherapy education which is based mostly on face-to-face teaching. Thus, educators have been compelled to adapt their pedagogical approaches moving to digital education. In this commentary, we debate on digital education highlighting its effectiveness, the users’ perspectives, and its weakness in the context of physiotherapy teaching aimed at informing post-COVID-19 future directions in this educational field. Existing evidence on digital education produced before COVID-19 supports its implementation into entry-level physiotherapy education. However, some challenges (e.g. social inequality and evaluation of students) threaten its applicability in post-COVID-19 era, calling educators to take appropriate actions.

## COVID-19 as a New Challenge for Physiotherapy Educators

The recent coronavirus disease 2019 (COVID-19) pandemic has challenged physiotherapy entry-level educational systems worldwide. Aimed at ensuring social distancing and physical isolation, governments have imposed restrictions on academic activities suspending and/or transforming teaching, workshops, and practice education [1]. Although these preventive measures have been implemented to curb the spread of COVID-19, they dramatically disrupted the routine of students and educators [1, 2]. With no possibility of delivering in-person taching [2], educators have been compelled to adapt their pedagogical approaches to digital education.

Digital education is an umbrella term reflecting the process of teaching and learning using information and communication technologies as a primary medium to connect students and educators who are physically separated (Table 1) [3–8].

**Table 1:** Differences between digital, traditional, and blended education. Reflections on benefits and challenges adapted from literature [3–18]

**Typology **	**Features**	**Benefits**	**Challenges**
Digital education	• *Definition*: the process of teaching and learning using digital technologies • *Modality*: contents are offered in digital format (e.g. PDF) or using digital technologies (e.g. computer-based digital interactions) • *Synonymous*: electronic learning, digital learning	• Increased accessibility	• Lack of application of learning theory or poor selection of learning theories for developing or supporting education (in curriculum design, program application, or learning evaluation)
		• Flexible access to learning content, no time or place limitation so student can learn anywhere and anytime	• Implementation restrictions caused by digital gap (requirement of technology infrastructure and digital literacy)
		• Abundant delivery Self-direction	• Additional development and set-up costs
		• Personalised learning experience	• High cost multimedia materials, high cost for platform maintenance, and require training for the user
		• Better sensation of content	• Untoward effects of digital education such as anxiety, dizziness, and isolation
		• Deeper information processing	
		• Adaptability	
		• Greater collaboration capacities	
		• Increased motivation	
		• Enjoyment of learning	
		• Cost-effectiveness	
		• Scalability that is possibility to increase or decrease size and requirements in response to changes	
		• Equity • Automatic evaluation and documentation of students’ progress, possibility of receiving feedback from the students • Ability to simulate and rehearse different clinical scenarios (experiential learning) • Interactive learning (didactic)	
		• Increased accessibility	
Traditional education	• *Definition*: the process of teaching and learning adopting non-digital materials (e.g. articles) or in-person human interaction (e.g. educators) • *Modality*: contents are offered both digitally (e.g. videos) and non-digitally (e.g. textbook) • *Synonymous*: classroom-based; face-to-face teaching; brick-and-mortar; in-ground	• Live demonstration of practice skills, followed by physical practice • Practice skills and get feedback from teacher • Synchronous delivery • Student interact in real-time Students experience a group dynamic • Students feel comfortable and learn more easily in a familiar, traditional classroom situation • Access more information and richer understanding through teacher and other students’ body language and voice	• Constraints in time space (classroom), and location
Blended education	• *Definition*: the process of teaching and learning which combines elements of traditional (e.g. in-person human interaction) and digital education (e.g. use of digital technologies) • *Modality*: contents are offered both digitally (e.g. online learning) and non-digitally (e.g. in-person human interaction) *• Synonymous*: hybrid	• Flexible access to learning content, student can learn anywhere and anytime	• Longer time on task compared to face-to-face learning
		• Provides learning needs of the students with various learning styles	• Expensive method of teaching (at least when a course is delivered for the first time)
		• Flexibility in learning	• Implementation restrictions caused by digital gap (requirement of technology infrastructure and digital literacy)
		• Lower order learning can be facilitated through online learning and onsite sessions focus on improving the higher order thinking of the learners (cover wider range and in-depth learning)	• Creation of necessary infrastructure
		• Self-direction	• High cost multimedia materials, high cost for platform maintenance, and require training for the user
		• Active learner’s participation	• Challenging to train faculty members in learning methods
		• Maximal utilisation of student learning time	Requires continuous blending of online and face-to-face sessions so the students realise the integration of the two components in a predetermined plan
		• Peer-learning	• Need of presence of teacher throughout online sessions, to provide timeline feedback and facilitate discussions — require specific skills
		• Enhanced engagement with peers and teachers	
		• Self-reflection	
		• Better learning experience	
		• By participating in online discussions and forums with their peers, learners gain the advantages of collaborative learning	
		• Improved the clinical reasoning skills	
		• More cost-effective for universities	
		• Fewer face-to-face sessions	

It includes a variety of digital modalities (e.g. computer-based digital education, mobile learning, and simulation-based education) [3–9] aimed to deliver contents either in real-time (e.g. synchronously) or pre-recorded (asynchronously) [10], providing the possibility of an Omni-Learning — anywhere, anytime, with anyone (Table 2) [11].

**Table 2:** Analysis of the most common digital technologies. Reflections on benefits and challenges adapted from literature [3–18]

**Typologies**	**Features**	**Benefits**	**Challenges**
Offline computer-based digital education	An educational strategy that needs no internet or local area network connection. It can be offered using media (e.g. CD-ROM, external hard disc, and flash memory)	• *Efficiency*: a wider variety of learning tools to deliver a more efficient learning experience making the learning process more effective, economical, and useful • *Access*: educators and learners alike gaining access to the platform in their own time and from a location of their choice. This should be considered for both offline and online access • *Cost*: cost-efficiency ratio varies according to the number of participants. Digital typologies that maximise attendance will result in an increased cost-efficiency • *Attendance*: this increases when students are able to follow a course remotely especially when in-person attendance is limited by geographical boundaries, transport constraints, or physical isolation guidelines (e.g. lockdown measures) • *Learning approaches*: as students are unique in their personal outlook and therefore gain from different learning styles, a diverse approach to learning will maximise the value for a more diverse student cohort and be more inclusive • *Scenarios*: virtual learning allows the possibility of multiple scenarios that would not be restricted by time in the in-person learning setting	• *Screentime*: Increasing the presence of screentime needs to consider issues related to focus, attention, and motivation, as well as a reduced level of physical activity • *Technology*: Poor or limited access to appropriate technology, inadequate technology, as well as its failure, will impact on the learning experience and process • *Human interaction*: isolated learning misses out on the interpersonal relationships that are created outside the formal learning environment • *Training needs*: educators and learners require special training to engage with digital technologies that may differ across different typologies, to ensure the correct use of it and optimise the outcome • *Affordability*: software programmes as well as access to appropriate technology have a cost • *Real-life interactions*: the absence of real-life settings (especially in practice-based classes) limits the experience of human interaction as well as the feedback necessary to optimise and maximise the learning event
Online computer-based digital education	An educational strategy delivered that uses “Internet Protocol” and a “Transmission Control Protocol” (e.g. online, web-based, and on a network)		
Serious gaming and gamification interventions	An educational strategy that applies competitive activities (e.g. games) or simulations (e.g. virtual environment) aimed to support students’ learning, cognitive, and practical skills		
Massive open online course (MOOC)	An educational strategy offered in form of an online course aimed to reach broad groups of geographically scattered students		
Virtual learning environment	An educational strategy that incorporates virtual environments to offer students the opportunity to experience specific learning activities in a non-physical world		
Virtual reality	An educational strategy that offers students the possibility of an active-learning experience in an immersive computer-generated environment (e.g. real or artificial)		
Virtual patient	An educational strategy that implies the adoption of an interactive computer simulation of healthcare scenarios aimed to develop students’ training, education, or assessment		
Digital psychomotor skills trainers	An educational strategy that adopts digital technologies (e.g. virtual reality) to improve students’ psychomotor skills (e.g. manual task)		
Mobile digital education (m-learning)	An educational strategy in which personal electronic devices (e.g. smartphone and tablet) are adopted to develop students’ learning and teaching beyond physical space and distance		

Digital education is not a new concept in entry-level physiotherapy education [12, 13]. Several systematic [14–16] and scoping reviews [17, 18] have investigated its effectiveness on learning processes and outcomes confirming that digital education has many benefits to offer. However, physiotherapy educators need to understand the role of digital technologies to address short-term educational issues posed by COVID-19 and to inform decisions for the post-pandemic time [19]. Moreover, despite efforts shared by different organisations [20] to adapt the educational system to the new reality (Table 3), consensus on practice standards and guidelines for digital education within physiotherapy curricula is still missing internationally [21].

**Table 3:** Example of resources for the physiotherapy community of educators

**International organisation**	**Resources**	**Sources**
World Physiotherapy	Education based resources	https://world.physio/covid-19-information-hub/covid-19-education-based-resources
International Neurological Physical Therapy Association	Teaching NPT in these crazy times: the entire podcast	https://inpaneurophysio.weebly.com/webinars.html
International Network of Physiotherapy Regulatory Authorities	Telehealth clinical education considerations	http://www.inptra.org/Webcasts.aspx
American Council of Academic Physical Therapy	ACAPT’s response to the COVID-19 “new normal”	https://acapt.org/news/news-detail/2020/04/29/acapt’s-response-to-the-covid-19-new-normal
European Network of Physiotherapy in Higher Education	Resources for educators — COVID-19 resources	https://www.enphe.org/resources-for-educators/

*COVID-19* coronavirus disease 2019, *NPT* Neurological Physical Therapy, *ACAPT* American Council of Academic Physical Therapy

Accordingly, this commentary aims to summarise and discuss existing evidence on digital education to highlight its strengths and weaknesses and the users’ perspectives in the context of physiotherapy teaching aimed to inform educators involved in physiotherapy as well as in other healthcare fields (e.g. nursing, speech therapy, occupational therapy, and medicine) on post-COVID-19 future directions. To this end, the commentary has been developed in accordance to the methodology suggested by Gasparyan et al. [22] as reported in Table 4.

**Table 4:** Methodology adopted for search and analysis. Reported from Gasparyan et al. [22]

**Typology**	**Details**
Sources accessed	• *Database*: MEDLINE through PubMed; Cumulative Index to Nursing and Allied Health Literature–CINAHL • *Other*: reference lists of pertinent articles
Search terms	• *Key-words*: physiotherapy, physical therapy, healthcare, students, undergraduate, entry-level, university; education, teaching, learning, simulation, digital, distance, web, social media, computer-assisted learning, multimedia, virtual, online, platform, video, COVID-19, coronavirus disease 2019, severe acute respiratory syndrome coronavirus 2, SARS-CoV-2 • *Boolean operators*: AND, OR
Limits	• *Time*: from inception of databases to 10th of January 2021 • *Language*: English, Italian
Studies included	• *Design*: quantitative (randomised controlled trial, pre-post) and qualitative (interview, focus groups) studies on the effectiveness and on the users’ perspective of digital education in physiotherapy • *Target*: entry-level physiotherapy students • *Topic*: adoption of any digital modalities for educational purposes • *Pubblication*: produced before and during COVID-19 pandemic
Steps for writing	• *Analysis*: collection, analysis, and organisation of findings, grouping of findings with similar data/level of evidence • *Reporting*: organisation of the main text into subsections, synthesis of findings into tables and figures, definition of major points for future research and practice, summary of new, evidence-based points

*CINHAL* Cumulative Index to Nursing and Allied Health Literature, *COVID-19* coronavirus disease 2019, *SARS-CoV-2* severe acute respiratory syndrome coronavirus 2

## The Effectiveness of Digital Education in Physiotherapy

Quantitative studies that analysed the effectiveness of digital education can be classified into three main categories: (a) those regarding the adoption of an online open-source platform; (b) those considering the use of online teaching; and (c) those about the different virtual learning experiences.

### Online Open-Source Platform

Open-source platforms have been used to provide physiotherapy students possibilities for peer consulting during their clinical practice or for providing knowledge on specific topics. In the two studies available, students participated in online peer consulting in gait analysis [23] and accessed an open-source platform for a module on spinal cord injuries [24] reporting significant increase in knowledge and confidence. In the module on spinal cord injuries [24], the confidence or satisfaction did not differ by the way they used the platform (work at their own pace or attend a massive online open course with guidance).

### Online Teaching

There are a few randomised controlled trials that have examined the effectiveness of *online teaching* in comparison with face-to-face teaching. Nicklen et al. [25] compared remote and face-to-face learning in case-based learning courses, where students collaborated in groups to solve a series of clinical problems. At post intervention, a multiple-choice test regarding the course content was administrated showing no significant differences between teaching modalities. Interestingly, students attending the remote learning group perceived that they did not reach the learning outcomes which may reflect some insecurity that students had with the remote modalities. Huhn et al. [26] compared two different teaching modalities: virtual (in an on-campus computer lab with faculty available to answer questions) and live (a large group discussion with faculty facilitator) showing no differences in the students’ clinical reasoning, knowledge acquisition, and transfer of knowledge. Two prospective, controlled, randomised, crossover studies [27, 28] where physiotherapy students participated in an oncology course [27] and in a basic course in psychomotor skills [28], using face-to-face classroom or e-learning, demonstrated similar results. In a study where teaching administration and management content in physiotherapy online were compared to face-to-face teaching, no significant difference in knowledge acquisition was found between the groups [29]. Moreover, in a course of scientific writing for healthcare students, including physiotherapist students, online instruction was better than standard face-to-face instruction in terms of writing quality [30]. Finally, a recent study produced during COVID-19 pandemic showed comparable students’ performance and satisfaction after a course delivered online when compared to an historic cohort of students who underwent the same course face to face in previous years [31].

### Virtual Learning Experiences

Several pre-post intervention studies have been conducted in which physiotherapy students participated in online virtual simulation courses of cultural empathy [32] and inter-professional collaboration [33, 34]. The results demonstrated significant improvements in the course topics taught supporting the use of online virtual environments in education. Furthermore, a study of the effectiveness of 360° video on students’ performance, satisfaction, and learning climate in an educational healthcare setting demonstrated that this video is equally effective compared to regular video but less effective than the face-to-face teaching [35]. Twogood et al. [36] described a new model for *virtual placement* that was tested after the UK entered a lockdown in the Spring of 2020 and then implemented across the Connect Health which is a physiotherapy service. The model combines shadowing a broad range of virtual clinics with the delivery of patient-facing online exercise classes via the Facebook Live platform and a completion of virtual projects to support knowledge consolidation. The outcome of this project was the number of students’ placements (the placement capacity) which increased 520% from the year 2019. This is the only study of online practice-based learning that has been published to our best knowledge. Both mentors and students were satisfied with the model. However, whether clinical physiotherapy standards are met by making use of a virtual placement requires further investigation.

## The Perception of Users Towards Digital Education

Qualitative studies analysing users’ experiences, perceived barriers, and facilitators towards digital education can be categorised within three broad domains: (a) those concerning the development of online education; (b) those considering the provision of online education; and (c) those around instruments used to support online education.

### Development of Online Education

Studies that investigated the development of online education [37–39] reported that educators appear to prefer a multicomponent approach that includes face-to-face teaching, hands-on skills learning in laboratories, the use of either electronic or hard copy materials, and online resources [37]. The advantage of using online learning was seen in the flexibility it offered towards time optimisation and accessibility of the teaching material [38]. However, students also expressed reluctance towards online learning as it was felt that this decreased social interaction [38]. The training in preparation of the online learning was also considered important as specific skills necessary to effectively engage in this form of learning could not be assumed to be present. The training was perceived to lead to an enhanced understanding of online learning with the result that resistance to change from face-to-face modalities diminished [38, 39].

### Provision of Online Learning

The provision of different types of online modules has been investigated and evaluated from the students’ perspective [33, 40–42]. Students stress the importance of the inclusion of scenarios and realistic simulations; especially real patients participating in the simulations [33, 41, 42]. This latter part was deemed necessary for the development of non-verbal communication skills, and as such, combining virtual learning with hands-on practice seems to be better indicated to address aspects of communication. Online learning was also perceived to overcome geographical barriers, bringing together students from different regions [40]. However, a suitable modality of allowing students’ interaction online, as during face-to-face education, as well as integrating strategies to facilitate online learning should be considered [42]. Motivation was considered an important aspect in online learning, and although controversial, the use of gamification was seen to enhance motivation especially in final course grades [41]. Technical difficulties, such as inconsistent system usability when multiple users logged in to the system simultaneously, were reported impacting negatively on the user experience [33]. However, we warrant caution when interpreting technical difficulties as a barrier to the provision of digital learning as firstly, it will depend on the type of online learning (e.g. online lecturing versus an integrated 3-D environment); and secondly, since developments in technology happen very rapidly, it is difficult to compare the resources available today to those available a few years ago.

### Online Instruments to Support Learning

Several studies have explored students’ perceptions of online instruments to support their learning such as learning repositories [43], video resources [44–46], and case-based online learning [47]. Whilst additional resources were recognised as a good basis for lifelong learning [43], a multicomponent learning resource was felt necessary for learning skills [44] (as in the case of physiotherapy education) with particular attention required for feedback [46]. Addressing technical issues such as a lack of technological prowess [46] including sound and video quality in the preparation of videos [44] was considered necessary, and better if targeted beforehand through appropriate training [47].

## Barriers of Digital Education that Emerged During COVID-19

**Fig. 1:**
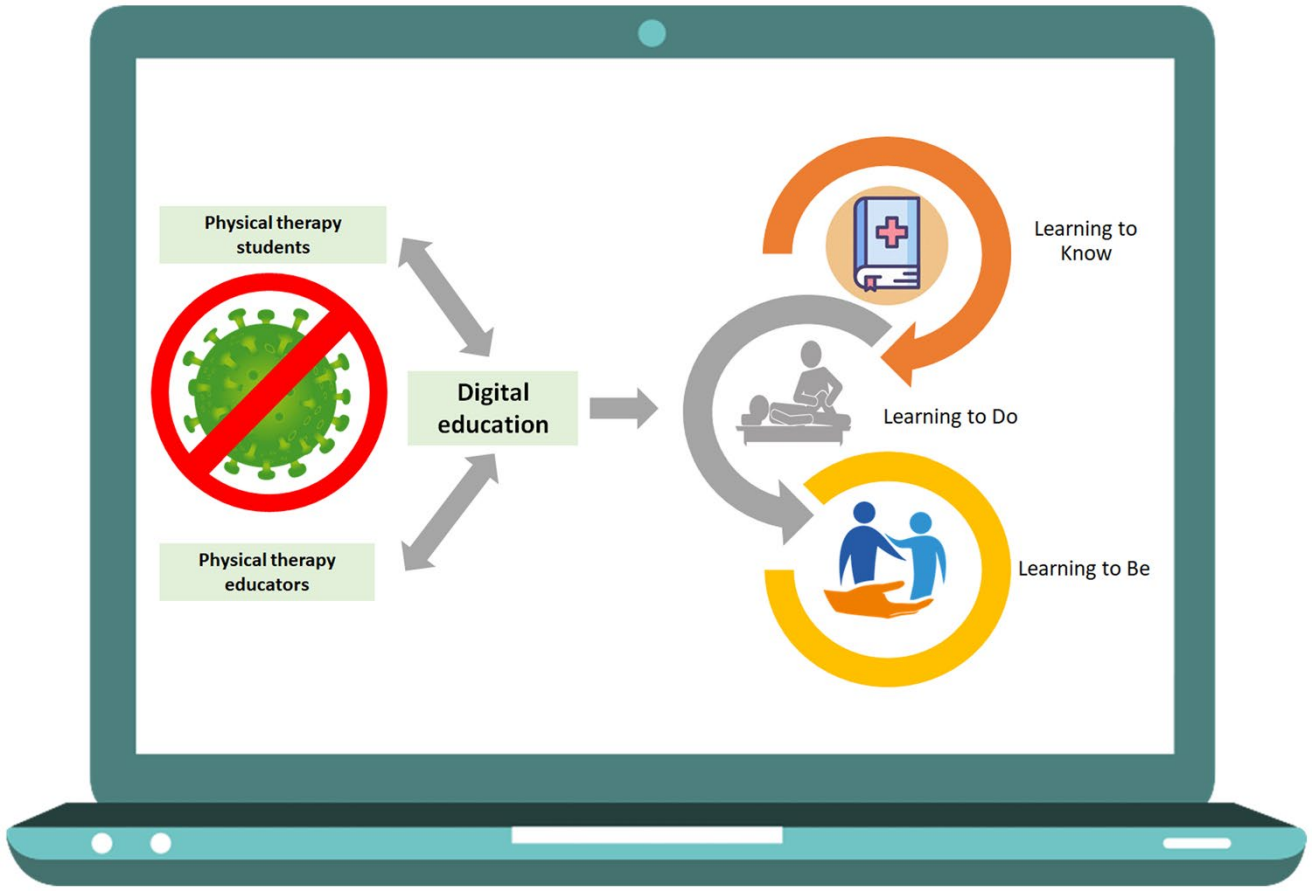
Opportunities of digital entry-level education in physiotherapy during COVID-19 pandemic and beyond. Legend: The image describes examples of students’ competences that can also be acquired using digital education as the theoretical (e.g. anatomical bony landmarks — “learning to know”), procedural (e.g. hand washing — “learning to do”), and relational ones (e.g. interaction with peers and educators — “learning to be”)

Whilst promising findings support the adoption of digital education in physiotherapy [12–18] (Fig. 1), caution should be adopted in generalising and transferring results to the current educational settings since data sampled from a non-emergency social context might not be applied to the context of the COVID-19 pandemic [19].

**Fig. 2:**
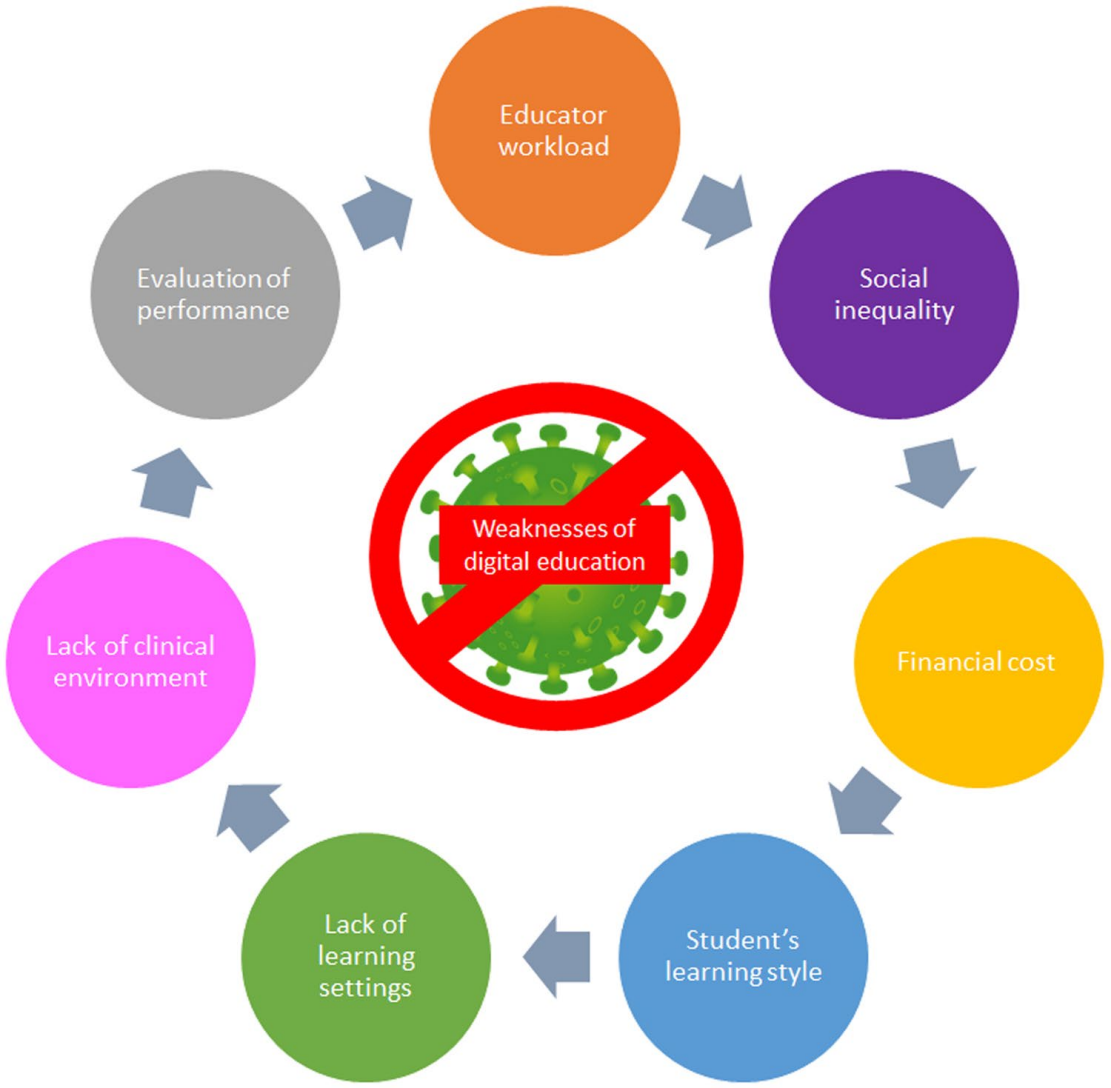
Weaknesses of digital entry-level education in physiotherapy during COVID-19 pandemic and beyond. Legend: The image displays a graphical summary of the weaknesses of digital education in physiotherapy as emerged from literature [48, 49]

Moreover, some critical issues emerged from interviews and focus groups on students [48] and educators [49] involved in entry-level physiotherapy education should be acknowledged as limitations of its applicability during COVID-19 pandemic and behind (Fig. 2).

### Unresolved Dilemmas

*Social inequality and digital divide* could limit students’ access to digital education. The lack of adequate digital resources (e.g. bandwidth connectivity, availability of hardware, and internet) constitutes a barrier to learning for those students living in rural regions and in developing countries [50]. There is a chance that some students who have not enough digital resources at home will not be able to cope with the contingent technology demand, affecting their learning outcomes [2, 49]. Moreover, universities could not have a financial budget or fail to obtain timely resources (e.g. software, platforms, and e-learning systems) for digital education, being unable to guarantee all students the same educational opportunities [49]. Such limitations, acting as a source of learning disparities, raise doubts about the sustainability of digital education.

The *financial cost of digital education* could impact students’ economic resources. In these uncertain times of COVID-19, the negative impact of the pandemic on the job market is a concern for students (e.g. loss of students’ part-time jobs) [1, 2, 48, 49], and could worsen the financial burdens in physiotherapy entry-level education which was already documented before the COVID-19 pandemic [51, 52]. Indeed, physiotherapy students have been reported to accumulate debts for studying at university, with the risk of failing to pay off loans [51, 52], thus limiting future lifelong learning choices and career advancements.

*Teaching style and learning material* offered digitally might not be aligned with the students’ preferred learning styles and educational needs. Although students should be able to use the different learning styles and channels (e.g. visual, kinesthetic, and auditory), they may prefer a specific learning style (e.g. kinesthetic) [53] which is difficult to ensure in digital education during COVID-19 for several technological limits (e.g. unavailability of resources for educators and students) [49]. Consequently, the difficulty of tailoring pedagogical materials to the students’ favoured learning styles could limit the acquisition and retention of knowledge [49], resulting also in a less motivating learning experience with increased risk of distractions (e.g. concomitant domestic activities, use of mobile phone, and engagement in social media) [48, 49]. Furthermore, as specific lectures (e.g. anatomy and physiology) can be conducted digitally, physiotherapy psychomotor skills (e.g. manual therapy and exercise laboratories) rely on the feedback of hands-on training and cannot be entirely replaced by digital modalities [1, 48, 49].

The contingent *lack of the physical learning settings* (e.g. classroom, desk, table, projectors, and skeleton) could impair the overall students’ learning experience. During digital education, various social cues are missing (e.g. interpersonal interactions among peers, real-time relationship with educators, and the atmosphere of the classroom) [48, 49], reducing the opportunity to develop a sense of belonging to a community of learners and to nurture cohesion and student’s group identity. Moreover, this shortage could threaten students’ well-being triggering psychological distress (e.g. irritability, fear, panic, avoidance behaviour, and anxiety) [2, 48, 49] registered among students during COVID-19 pandemic.

The *absence of practice education* in the clinical environments reflects an important vacuum in the physiotherapy curriculum. Lack of direct therapeutic interactions with patients, mentors, and experienced clinical educators represent missed learning experiences, compromising the development of the student’s professional and personal skills, attitudes, and behaviours (e.g. learning to know, learning to do, and learning to be) [1, 48, 49]. This emergency could limit the opportunity to expose students to various clinical settings (e.g. inpatients, outpatients, public, and private), diseases and conditions (e.g. acute, subacute, and chronic), areas of physiotherapy (e.g. musculoskeletal, respiratory, geriatric, neurological, and urogynecological), limiting also the chance to network with peers involved in interdisciplinary teams (e.g. nursing, medicine, and speech therapy) [54].

The *evaluation of students’ performance* could be superficial in digital education. Challenges to perform practical examination (e.g. performance test for technical and nontechnical skills), and to identify a shared assessment format (e.g. dichotomous score/pass-fail or continuous score/number or letter grading system) [1, 49] all represent difficulties that could threaten solid, integral, and secure evaluations, aggravating the existing problems of evaluation (e.g. poor psychometric — validity and reliability — and edumetric properties — feasibility, usefulness, and educational impact — of assessment tools) in physiotherapy education [55]. Moreover, educators should not underestimate that a new unfamiliar model of assessment, as well as the lack of vigilance during the exam performed at home, could lead students to academic misconduct (e.g. fraud, cheating, and hint) [56], increasing the risk to replicate this unsuitable behaviour as a clinician in future, thus undermining the grow of a health professional’s culture based on ethical and deontological values (e.g. integrity, honesty, and responsibility).

Finally, *the workload required to prepare digital teaching* could overwhelm educators. The high workload, as well as the pressures to quickly produce high-quality didactic resources, the need to perform other concomitant academic duties (e.g. publishing research, administration, and clinical service), and the difficulty to determine the boundaries of professional and personal activities [1, 49] all represent challenges for the psychological well-being of educators and possible sources of burnout and technophobia. In addition, educators are needed to receive further training to develop a different competency framework for online teaching and assessment and familiarise themselves with different pedagogical model and digital teaching platforms [49].

### Future Perspectives of Digital Education

At first glance, the COVID-19 pandemic would appear to be a substantial problem in physiotherapy education [48, 49]. However, the challenges being faced in these extraordinary times have offered the opportunity to rethink education systems extensively, resulting in an overall advancement of physiotherapy and calling to action the stakeholders involved in the entry-level education processes (e.g. institutions, educators, students, and researchers) (Table 5).

**Table 5:** Suggested actions needed at multiple levels of entry-level education in physiotherapy to counteract dilemmas of digital education outlined in “Unresolved dilemmas” section

**Levels**	**Actions needed**	**Target**
Institution	(a) To analyse cost-benefits of digital education, considering economic and organisational issues (e.g. reimbursement systems, revision of university taxes, and planning extracurricular activities);	2
	(b) To identify problems (e.g. unstable internet connections) and shortages of resources (e.g. lack of laptops and electronic devices) encountered by students, guaranteeing equity of education;	1
	(c) To offer educators resources to cope with digital education (e.g. access, training, and support), considering the time required by the learning curve to acquire adequate education not only using new technology but also developing a completely different competency framework for teaching and assessment;	7
	(d) To deliver an updated, opened, reliable, transparent, and frequent communication (e.g. phone, email, institutional website, and questions & answers sessions), reassuring students and staff (e.g. analyse their emotional and psychological distress and improve their motivation and engagement);	4
	(e) To introduce in learning curriculum COVID-19 topics (e.g. prevention and adoption of personal protective equipment), preparing students for safe clinical experiences	5
Educators	(a) To create effective (e.g. avoid slides heavily text-based and prefer images and animations) and efficient teaching sessions (e.g. summarise learning objectives and lessons no excessively long), capable of ensuring a high quality of digital education in terms of consistency and structure of the course;	3
	(b) To adopt digital methods of teaching (e.g. online) aligned with students’ learning style, implementing both synchronous (e.g. livestream discussion, teleconferencing, webinars, and real-time lectures) and asynchronous (e.g. links for reading, short quiz, e-learning platform, case studies, recorded lectures, and animations) tools, facilitating questions & answers with students;	3
	(c) To offer alternative practice education both clinically and laboratory-based, sharing with students digitally the management of patients in virtual rooms (e.g. history taking, physical examination, decision-making, therapy administration, physiotherapy program, teleconsultation and telerehabilitation; and video clinical vignettes) providing feedback and guidance;	5
	(d) To use sophisticated evaluation systems (e.g. randomised questions and “live” examinations), aligning tools with educational contents delivered during digital education;	6
	(e) To implement collaborations and learning among peers activating digitally small groups of work with different levels of complexity (e.g. team-based learning, small group case-based or problem-based learning) supporting different types of interactions (e.g. encouraging “live” discussion and chat conversation; sharing laptop screens, resources, papers and notes);	4
	(f) To be trained in order to develop new skills and competencies aimed at offering qualitative digital education regarding teaching and assessment (e.g. lifelong learning)	7
Researchers	(a) To run studies on effectiveness of digital education with high methodological quality (e.g. large sample size, validated and homogeneous outcomes measures), including the point of view of students and educators (e.g. satisfaction, experience, and strength and weakness);	1–7
	(b) To perform research at an international level, involving all the different world regions (e.g. Asia Western Pacific, Africa, North America Caribbean, South America, and Europe), including students from all university levels (from 1st year to PhD), considering education with different backgrounds (e.g. novice and experts);	1–7
	(c) To implement investigation on virtual reality technologies (e.g. digital, immersive, augmented environments), involving visual and haptic feedback useful for the development of different competences (e.g. clinical reasoning, hands-on skills, and therapeutic relationship);	1–7
	(d) To measure the effects of digital education, including analyses at multiple levels (e.g. pedagogical, organisational, cost-effectiveness, and well-being/social);	1–7
	(e) To consider long-term outcomes of digital education, analysing its future impact on clinical performance (e.g. during practice education) and choice of professional career (e.g. work in inpatients or outpatient setting)	1–7
Students	(a) To report any specific learning disabilities (e.g. dysgraphia, dyslexia, and dyscalculia), impairments (e.g. low vision and hearing loss), or other neurodevelopment disorders (e.g. communication disabilities and attention-deficit/hyperactivity disorders), threatening the learning process and outcomes through the use of digital education;	1
	(b) To collaborate in the quality improvement processes based upon surveys (e.g. local, national, and international), where the collections of data are aimed at monitoring the implementation and the outcomes of digital education in physiotherapy;	1,2
	(c) To inquire about university initiatives aimed at providing free guidance tools for the use of digital education (e.g. Power Point presentation, documents, and video), facilitating their overall learning;	3,4
	(d) To act as a self-direct learner by searching for resources aimed at discovering new frontiers of digital education (e.g. podcasts, blogs, webinars, virtual journal club, high-quality YouTube™ videos, and massive open online courses), developing new skills useful both for their current training and for their lifelong learning;	3,4
	(e) To create and nurture a community network among peers and with students from other universities using chat and social media (e.g. Facebook™, Twitter™, and Instagram™), sharing experiences about the strengths and limitations of digital education as well as starting to act as a future community of physiotherapists	

*COVID-19* coronavirus disease 2019, *PhD* doctor of philosophy, *1* social inequality and digital divide, *2* financial cost, *3* misalignment of teaching style and learning material, *4* lack of the physical learning settings and resources *5* absence of practice education *6* superficiality of students’ evaluation, *7* workload required to prepare digital teaching

Despite both strengths and weaknesses of digital education in physiotherapy being extensively reported [12–18], this unpreceded emergency has given the physiotherapy community the opportunity to further reflect on the value of digital education [1, 57].

Although the efforts to tackle the COVID-19 pandemic are shared among entry-level educational programs in physiotherapy worldwide [1, 2, 20], deviations in management are expected due to variations of federal, local, and national policies, the spread of the virus, and the availability of resources. Therefore, there is a need to pursue common goals, balancing the health of students, educators, and patients, whilst ensuring the continuity of education, the certification of competence, and the resilience to continue learning and teaching digitally.

Despite the still unknown long-term impact of COVID-19 pandemic on physiotherapy education [1] and the actual shortage of evidence on risks and benefits of digital education produced during COVID-19 [48, 49], re-engineering the negative educational events to embrace a positive learning experience will enable the upskill of new technologies. Thus, a culture of digital teaching and learning should be encouraged [58] to prepare the institutions for a “new” educational system that would be adapted to other unforeseen situations in the future.

Moreover, aligning the teaching of students’ skills and competencies with the progress of technology and reviewing didactic contents will facilitate the digital transformation of entry-level physiotherapy education contributing to preparing digitally enabled and literate future physiotherapists (e.g. stimulating self-directed and autonomous learning using online contents) [21], and creating wider academic communities of researchers capable to share new ideas, projects, and experiences applicable in different educational contexts (e.g. as happened during the 5th European Congress on Physiotherapy Education of the European Region World Physiotherapy) [59].

## Conclusion

The wind of change is blowing, leaving the innovations knocking at the door. When the COVID-19 pandemic will be over, looking back on this experience, we are confident that the chances offered will exceed the difficulties faced because the deeper the challenges, the greater the advancements. As educators involved in physiotherapy programmes, we have the responsibility to act together thinking inside and outside the box, to embrace flexible and adaptive operational modalities moving away from our comfort zone to ensure a high level of competencies for the future generation of physiotherapists. In summary, digital education can be applied in physiotherapy as a resource in replacement (e.g. for theoretical knowledges — online) or in integration (e.g. for procedural skills — blended) of the face-to-face teaching. This commentary is aimed at encouraging all educators involved in the different healthcare fields (e.g. nursing, speech therapy, occupational therapy, and medicine) to consider the digital education within their entry-level educational programs.
